# Early-Life Soy Exposure and Gender-Role Play Behavior in Children

**DOI:** 10.1289/ehp.1103579

**Published:** 2011-08-03

**Authors:** Margaret A. Adgent, Julie L. Daniels, Lloyd J. Edwards, Anna Maria Siega-Riz, Walter J. Rogan

**Affiliations:** 1 Department of Epidemiology, Gillings School of Global Public Health, University of North Carolina–Chapel Hill, Chapel Hill, North Carolina, USA; 2 Epidemiology Branch, National Institute for Environmental Health Sciences, National Institutes of Health, Department of Health and Human Services, Research Triangle Park, North Carolina, USA; 3 Department of Biostatistics, Gillings School of Global Public Health, University of North Carolina–Chapel Hill, Chapel Hill, North Carolina, USA

**Keywords:** ALSPAC, endocrine disruptor, gender, infant formula, isoflavone, play behavior, PSAI, sexual dimorphism, soy

## Abstract

Background: Soy-based infant formula contains high levels of isoflavones. These estrogen-like compounds have been shown to induce changes in sexually dimorphic behaviors in animals exposed in early development.

Objective: We examined gender-role play behavior in relation to soy-based and non-soy-based infant feeding methods among children in the Avon Longitudinal Study of Parents and Children.

Methods: We studied 3,664 boys and 3,412 girls. Four exposure categories were created using data from questionnaires administered at 6 and 15 months postpartum: primarily breast, early formula (referent), early soy, and late soy. Gender-role play behavior was assessed using the Pre-School Activities Inventory (PSAI). Associations between infant feeding and PSAI scores at 42 months of age were assessed using linear regression. Post hoc analyses of PSAI scores at 30 and 57 months were also conducted.

Results: Early-infancy soy use was reported for approximately 2% of participants. Mean [95% confidence interval (CI)] PSAI scores at 42 months were 62.3 (62.0, 62.6) and 36.9 (36.6, 37.2) for boys and girls, respectively. After adjustment, early soy (vs. early formula) feeding was associated with higher (less feminine) PSAI scores in girls (® = 2.66; 95% CI: 0.19, 5.12) but was not significantly associated with PSAI scores in boys. The association between soy exposure and PSAI scores in girls was substantially attenuated at 30 and 57 months.

Conclusions: Although not consistent throughout childhood, early-life soy exposure was associated with less female-typical play behavior in girls at 42 months of age. Soy exposure was not significantly associated with play behavior in boys.

Soy-based infant formula (SBF) is a commonly used alternative to cow milk–based infant formula, accounting for 12–20% of the infant formula sold in the United States and 2–7% in the United Kingdom (Bhatia et al. 2008; Committee on Toxicity of Chemicals in Food 2003; Essex 1996; National Toxicology Program 2010). Although SBF is considered to be nutritionally adequate for term infants (Bhatia et al. 2008), the overall safety of SBF has recently been debated because it contains high levels of the isoflavone compounds genistein and daidzein, plant-based compounds with structural and functional similarity to the steroid hormone 17®-estradiol (“phytoestrogens”) (Badger et al. 2009; Barrett 2002; Chen and Rogan 2004; Setchell et al. 1997; Tuohy 2003; Vandenplas et al. 2010). These compounds can bind to estrogen receptors (ERs) with an affinity that is 100–1,000 times less than that for estradiol and at least an order of magnitude higher than that for the industrial endocrine-disrupting compound bisphenol A (Kuiper et al. 1998). Infants on an SBF diet have extremely high exposures to soy isoflavones, with urine isoflavone concentrations approximately 500 times those in cow milk formula–fed infants (Cao et al. 2009), but the effects of isoflavone exposure in the postnatal period on child development are largely unknown. It is important to explore how early-life exposures to soy products may affect hormonally driven developmental outcomes.

Sexually dimorphic brain development and behavior are influenced by steroid hormones in the prenatal and early postnatal periods (Arai et al. 1996; Davis et al. 1996; reviewed by Cohen-Bendahan et al. 2005; Dickerson and Gore 2007); thus, assessing the effects of early-life soy exposures on these end points is of great interest. Accordingly, animal models have demonstrated that sexually dimorphic outcomes are sensitive to soy isoflavones. For instance, the volume of sexually dimorphic regions of the brain (Faber and Hughes 1991, 1993; Lund et al. 2001a; Scallet et al. 2004), in addition to other dimorphic traits such as visual spatial memory (Lephart et al. 2002; Lund et al. 2001b) and mating behaviors (Kouki et al. 2003; Wisniewski et al. 2003), has been shown to be affected by phytoestrogen exposures in male and female rats across a range of doses and life stages, albeit somewhat inconsistently (Lewis et al. 2003; Masutomi et al. 2003; Patisaul et al. 2007).

Human studies have similarly indicated that sexually dimorphic behaviors are susceptible to early-life hormone exposures, but little is known about the effects of postnatal estrogens. High prenatal androgen exposures have consistently been associated with girls’ preference for male-typical toys and interests in several studies of girls with congenital adrenal hyperplasia (Berenbaum and Hines 1992; Berenbaum et al. 2000; Iijima et al. 2001) and in studies of maternal and amniotic testosterone (Auyeung et al. 2009; Hines et al. 2002a). Recent studies suggest that postnatal testosterone may also be associated with male-typical development, as measured by sexually dimorphic auditory processing (Friederici et al. 2008) and social preferences in boys (Alexander et al. 2009).

The postnatal period is a time of elevated hormonal activity (Andersson et al. 1998; Chellakooty et al. 2003), and the hypothalamus, which is integral in postnatal hormone regulation via the hypothalamic–pituitary–gonadal (HPG) axis, has a concentration of ERs (Osterlund et al. 2000). It is plausible that ER binding to isoflavones may interfere with normal HPG axis function and, subsequently, disrupt regulated postnatal hormonal concentrations. Therefore, exploring the effects of postnatal soy exposures on sexually dimorphic development is warranted.

Observing gender-role play behaviors is a convenient method for assessing sexual dimorphism in young children. Characterized by a child’s preference for certain masculine- or feminine-typical toys, activities, and attitudes, gendered play behavior may be present as early as 12 months of age and typically becomes increasingly sex specific with age (Golombok et al. 2008; O’Brien and Huston 1985; Servin et al. 1999; Snow et al. 1983). These characteristics, as measured by instruments such as the Pre-School Activities Inventory (PSAI), have been used to assess sexually dimorphic behaviors in children in previous studies of environmental endocrine disruptors, including phthalates, polychlorinated biphenyls, and dioxins (Swan et al. 2010; Vreugdenhil et al. 2002). Here, we assessed the association between gender-role play behavior and infant feeding methods, with particular interest in the effects of early-life soy exposure.

## Materials and Methods

*Study sample.* Women who were pregnant, residing in the Avon region of the United Kingdom, and expected to deliver between 1 April 1991 and 31 December 1992 were eligible for the Avon Longitudinal Study of Parents and Children (ALSPAC); 14,062 pregnancies were recruited into the study that resulted in live births, and of these, 13,978 were twins or singletons alive at 1 year. The present investigation was restricted to term singletons (*n* = 12,931) for whom complete infant feeding data were available (*n* = 8,519) and for whom a play behavior outcome assessment was completed at approximately 42 months of age. The total study sample was 7,076 participants (3,664 boys and 3,412 girls). Mothers provided informed consent for participation. Ethical approval for the study was obtained from the ALSPAC Law and Ethics Committee and the Local Research Ethics Committees. The present analysis was approved by the Institutional Review Board of the University of North Carolina–Chapel Hill.

*Exposure assessment.* Mothers completed infant feeding questionnaires at 1, 6, 15, and 24 months postpartum. Mothers reported current breast-feeding habits, the age at which other milks or formulas were introduced into the child’s diet (including formula/baby milk, soy milk, soy formula, goat’s milk, follow-on milk, hypoallergenic formula, and cow’s milk), and how many feedings per week were given for each of these products at the time of questionnaire completion.

We defined exposure categories using responses to the questionnaire administered at 6 months postpartum; if these data were missing or incomplete, responses from the 15-month questionnaire were used. “Early” exposure was defined as the use of a specific formula or milk type occurring ≤ 4 months of age [“At what age did you start (formula/milk type)?”] through ≥ 6 months of age. Use at 6 months was indicated by any nonzero response to the question “How often nowadays is your baby fed (formula/milk type)?” in the 6-month questionnaire. If the 15-month questionnaire was used instead, “early” exposure to formula was established for any participant that reported introducing the formula or milk ≤ 4 months of age and responded affirmatively to the question “Since your child was 6 months old, has he/she had (formula/milk type)?” This definition established not only early use of formula but also a 1-month minimum duration of use.

**Figure 1:**
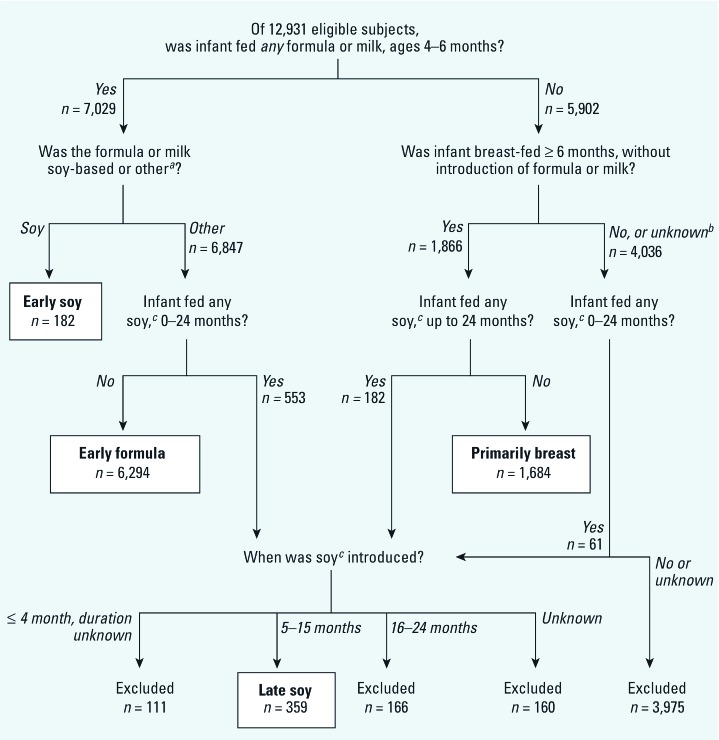
Exposure characterization. Participants were classified into four mutually exclusive feeding groups (primarily breast, early formula, early soy, late soy). Participants who did not meet an exposure definition or who had incomplete feeding data were excluded. ***^a^***Other: formula/baby milk, goat’s milk, follow-on milk, hypoallergenic formula, and cow’s milk. ***^b^***Unknown: data insufficient to determine breast-feeding duration or milk/formula use through 6 months. ***^c^***Soy: soy milk or soy formula.

We categorized participants into four mutually exclusive feeding groups: primarily breast-fed, early formula, early soy, and late soy (Figure 1). Primarily breast-fed infants were those who were breast-fed until ≥ 6 months of age who had no reported introduction of other milks or formulas before 6 months of age and no reported soy milk/formula use before 24 months of age. Early formula-fed infants were those introduced to any nonsoy milk or formula product ≤ 4 months of age, had sustained use of such products at 6 months of age, and no reported soy use before 24 months of age. Early soy-fed infants were those introduced to soy milk or soy formula ≤ 4 months of age who had sustained use at 6 months of age. Late soy-fed infants were those introduced to soy milk or soy formula any time after 4 months of age through 15 months of age. We did not restrict the early formula, early soy, and late soy groups with respect to duration of breast-feeding; likewise, we did not restrict the early or late soy groups with respect to use of nonsoy formula.

We excluded participants if feeding profiles were not sufficiently complete to estimate duration of a particular feeding method or if their known exposure profile did not fit into an exposure classification. We also excluded participants who reported soy use only between 15 and 24 months because we assumed that exposure would be low compared with earlier times in infancy. Responses from the 1-month questionnaire were used to verify that no soy was used in early infancy among primarily breast-fed, early formula, and late soy participants. Exposure definitions did not take into account exposure to solid foods or their corresponding soy content, if any.

*Outcome assessment.* We used the PSAI, a psychometric test designed to assess within and between gender differences in early-life play (Golombok and Rust 1993a, 1993b), to assess gender-role play behavior. To complete the PSAI, mothers or other primary caregivers reported how often their child had played with certain toys (7 items), engaged in certain activities (11 items), and displayed certain characteristics (6 items) for the past month. Half of these items were “masculine,” and half were “feminine.” Each response was scored on a 5-point Likert scale, ranging from “never” to “very often.” The instrument was scored by summing responses to masculine items, subtracting the sum of feminine items, and applying a transformation (48.25 + 1.1 × score) to achieve a “pseudo–*t*-score” (Golombok and Rust 1993b). Higher scores indicate masculine typical behavior, and lower scores indicate feminine typical behavior.

PSAI assessments were administered in ALSPAC at 30, 42, and 57 months of age. We chose the 42-month assessment as our primary outcome of interest, before any analysis, because this age was similar to the age of the sample within which the test was validated (Golombok and Rust 1993b) and because this time point has been used in previous PSAI publications (Hines et al. 2002a, 2002b; Rust et al. 2000). In post hoc analyses, we used the 30- and 57-month assessments to evaluate consistency of associations over time.

*Covariates.* Demographic, family composition, and lifestyle characteristics were assessed through parent report on self-completed questionnaires. The mother’s and partner’s interaction with the child was estimated when the child was 42 months of age using a series of questions assessing the frequency at which each parent participated in a list of eight activities with the child (score range, 0–36). Partner interaction scores were set to zero if the questionnaire reported that no partner was present. We assumed partners were male, given a low prevalence of mothers in same-sex partnerships in this cohort (< 1%) (Golombok et al. 2003).

*Analysis.* Crude mean PSAI scores were assessed as means with 95% confidence intervals (CIs) within exposure groups and within strata of each covariate. Adjusted mean differences in PSAI scores were estimated using multivariable linear regression. Boys and girls were modeled separately to distinguish within-sex differences. The early formula-feeding group was used as the referent. We identified possible confounders as variables thought to be associated with gender role development and statistically associated with any infant feeding method in univariable investigations of these data (chi-square or *t*-test, *p* < 0.05, compared with early formula referent). Final models were adjusted for age at assessment, presence of an older brother (yes/no) or an older sister (yes/no), regular child-care attendance (yes/no), maternal and partner interaction scores, and maternal factors, including age at delivery, smoking in the third trimester (yes/no), and education [ranked from high to low: university degree, advanced level, ordinary level, vocational, and Certificate of Secondary Education (CSE)/none].

Data were analyzed using both complete case analysis and multiple imputation. For the complete case analysis, participants with missing data for adjustment variables (~ 18%) were dropped from models, so only true values for participants with complete data were modeled. All analyses were completed using SAS (versions 9.1.3 and 9.2; SAS Institute Inc., Cary, NC). For multiple imputation models, values for missing covariates were estimated from available data on all adjustment variables, as well as breast-feeding duration and marital status, using PROC MI (five iterations). Regression models of imputed data were run and summarized in PROC MIANALYZE.

## Results

The characteristics of our study sample (*n* = 7,076) and of all term singleton ALSPAC births (*n* = 12,931) are shown in Table 1. When we compared our study sample with participants excluded on the basis of insufficient data (*n* = 5,855; data not shown), included and excluded participants were similar with respect to sex, feeding method, and PSAI score distribution (among nonmissing). However, the included sample had fewer children with older brothers (32.2% vs. 36.3%), older sisters (30.8% vs. 33.7%), and mothers who smoked (16.8% vs. 24.0%) and more children with mothers holding a university degree (15.1% vs. 11.7%), slightly older mothers (mean maternal age, 28.7 years vs. 27.1 years), and better partner interaction (mean scores, 20.3 vs. 19.7) than did participants that were excluded (proportions do not account for missing data). Among the included participants, approximately 37% attended child-care regularly. Most mothers were nonsmokers during the prenatal period and had mid- to high-level education. Approximately 6% of households reported the absence of a partner.

**Table 1:** Characteristics of study sample and eligible ALSPAC participants.

Study sample				
Characteristic	Boys (*n* = 3,664)	Girls (*n* = 3,412)	Total (*n* = 7,076)	ALSPAC*a *(*n* = 12,931)
PSAI score (mean ± SD)		62.3 ± 8.6		36.9 ± 9.3		50.1 ± 15.5		50.0 ± 15.6
Missing (*n*)		—		—		—		3,580
Age at PSAI completion [months (mean ± SD)]		42.3 ± 0.8		42.3 ± 0.8		42.3 ± 0.8		42.3 ± 0.9
Missing (*n*)		80		83		163		3,809
Maternal age [years (mean ± SD)]		28.9 ± 4.7		28.6 ± 4.6		28.7 ± 4.7		28.0 ± 5.0
Missing (*n*)		0		0		0		0
Mother interaction score (mean ± SD)		28.5 ± 4.9		28.8 ± 4.7		28.6 ± 4.8		28.6 ± 4.8
Missing (*n*)		8		3		11		3,597
Partner interaction score (mean ± SD)		20.4 ± 7.9		20.1 ± 8.1		20.3 ± 8.0		20.1 ± 8.1
Missing (*n*)		15		22		37		3,638
Partner absent [*n* (%)]		216 (5.9)		219 (6.5)		435 (6.1)		609 (6.6)
Infant feeding method [*n* (%)]								
Early formula		2,697 (73.6)		2,488 (72.9)		5,185 (73.3)		6,294 (73.9)
Early soy		89 (2.4)		68 (2.0)		157 (2.2)		182 (2.1)
Late soy		167 (4.6)		139 (4.1)		306 (4.3)		359 (4.2)
Primarily breast-fed		711 (19.4)		717 (21.0)		1,428 (20.2)		1,684 (19.8)
Missing (*n*)		—		—		—		4,412
Presence of older brother [*n* (%)]								
No		2,344 (66.6)		2,261 (69.1)		4,605 (67.8)		6,816 (66.4)
Yes (≥ 1)		1,175 (33.4)		1,010 (30.9)		2,185 (32.2)		3,443 (33.6)
Missing (*n*)		145		141		286		2,672
Presence of older sister [*n* (%)]								
No		2,399 (68.2)		2,294 (70.2)		4,693 (69.2)		6,991 (68.2)
Yes (≥ 1)		1,118 (31.8)		976 (29.8)		2,094 (30.8)		3,264 (31.8)
Missing (*n*)		147		142		289		2,676
Regular child care attendance [*n* (%)]								
No		2,229 (64.3)		2,017 (62.3)		4,246 (63.3)		5,943 (63.0)
Yes		1,240 (35.7)		1,223 (37.7)		2,463 (36.7)		3,490 (37.0)
Missing (*n*)		195		172		367		3,498
Prenatal smoking [*n* (%)]								
No		2,924 (82.7)		2,769 (83.7)		5,693 (83.2)		8,898 (80.5)
Yes		611 (17.3)		539 (16.3)		1,150 (16.8)		2,162 (19.5)
Missing (*n*)		129		104		233		1,871
Maternal education [*n* (%)]								
University degree		505 (14.7)		498 (15.6)		1,003 (15.1)		1,497 (13.8)
Advanced level		865 (25.2)		826 (25.8)		1,691 (25.5)		2,607 (24.1)
Ordinary level		1,316 (38.4)		1,195 (37.4)		2,511 (37.9)		4,002 (36.9)
Vocational		337 (9.8)		297 (9.3)		634 (9.6)		1,132 (10.5)
CSE/none		406 (11.8)		379 (11.9)		785 (11.9)		1,596 (14.7)
Missing (*n*)		235		217		452		2,097
**a**Eligible ALSPAC participants are limited to term, singleton infants alive at 1 year.								

**Figure 2:**
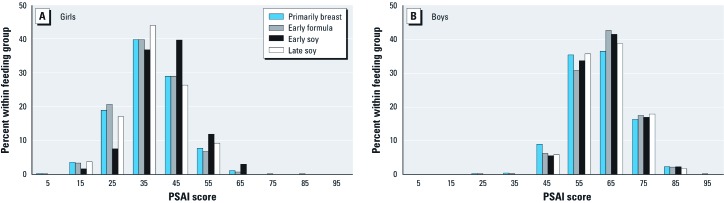
Distribution of 42-month PSAI Scores by sex and feeding group for (*A*) girls and (*B*) boys.

Unless otherwise specified, all results pertain to the 42-month PSAI assessment. Boys’ and girls’ PSAI scores had distinct normal distributions [Figure 2; mean (95% CI), range: boys, 62.3 (62.0, 62.6), 20.8–95.6; girls, 36.9 (36.6, 37.2), 4.3–85.7] and were similar to scores previously reported in this cohort (Golombok et al. 2008; Hines et al. 2002b). The PSAI assessment was completed between 41–53 months of age for boys and 41–54 months of age for girls. Early soy-fed infants accounted for 2.4% of boys and 2.0% of girls.

Among girls, the mean PSAI score [mean (95% CI)] was highest (less feminine) with early soy feeding [40.8 (38.6, 43.0)] and lowest with early formula feeding [36.7 (36.4, 37.1)] (Table 2). Among boys, PSAI scores were lowest (less masculine) in the primarily breast-fed group [61.3 (60.6, 61.9)] and highest in the early soy-fed group [63.0 (61.3, 64.7)]. However, early soy feeding in boys was not associated with a mean score that was significantly different from that of the early formula-fed boys [62.6 (62.2, 62.9)]. No significant difference was observed between late soy and early formula feeding for boys or girls or between breast-feeding and early formula feeding for girls.

**Table 2:** Crude mean PSAI scores and regression estimates (β) for exposure groups and select categorical and continuous covariates.

Covariate	Boys	Girls
Infant feeding method [mean PSAI score (95% CI)]				
Early formula*a*		62.6 (62.2, 62.9)		36.7 (36.4, 37.1)
Early soy		63.0 (61.3, 64.7)		40.8 (38.6, 43.0)
Late soy		62.1 (60.9, 63.3)		37.0 (35.4, 38.6)
Primarily breast-fed		61.3 (60.6, 61.9)		37.1 (36.4, 37.8)
Presence of older brother [mean PSAI score (95% CI)]				
No*a*		61.6 (61.2, 61.9)		35.9 (35.5, 36.3)
Yes (≥ 1)		63.7 (63.2, 64.2)		39.2 (38.7, 39.8)
Missing		62.8 (61.4, 64.1)		36.2 (34.6, 37.7)
Presence of older sister [mean PSAI score (95% CI)]				
No*a*		63.2 (62.9, 63.6)		37.5 (37.2, 37.9)
Yes (≥ 1)		60.2 (59.7, 60.8)		35.5 (34.9, 36.1)
Missing		62.7 (61.3, 64.0)		36.2 (34.7, 37.7)
Regular child care attendance [mean PSAI score (95% CI)]				
No*a*		62.2 (61.9, 62.6)		36.7 (36.3, 37.1)
Yes		62.4 (61.9, 62.9)		37.2 (36.7, 37.8)
Missing		62.7 (61.4, 64.0)		37.1 (35.7, 38.4)
Prenatal smoking [mean PSAI score (95% CI)]				
No*a*		62.0 (61.7, 62.3)		36.7 (36.3, 37.0)
Yes		63.7 (63.0, 64.4)		38.2 (37.4, 39.1)
Missing		62.0 (60.5, 63.6)		36.2 (34.5, 37.9)
Maternal education [mean PSAI score (95% CI)]				
University degree*a*		60.5 (59.8, 61.3)		38.6 (37.8, 39.4)
Advanced level		62.0 (61.4, 62.6)		37.4 (36.7, 38.0)
Ordinary level		62.7 (62.2, 63.1)		36.2 (35.6, 36.7)
Vocational		62.4 (61.6, 63.3)		35.9 (34.9, 36.9)
CSE/none		63.2 (62.4, 64.1)		36.9 (36.0, 37.9)
Missing		63.2 (62.1, 64.4)		36.6 (35.3, 37.8)
Age at PSAI assessment [β (95% CI)]		0.24 (–0.12, 0.60)		–0.46 (–0.85, –0.08)
Maternal age at delivery [β (95% CI)]		–0.02 (–0.08, 0.04)		0.17 (0.11, 0.24)
Maternal interaction score [β (95% CI)]		0.02 (–0.04, 0.08)		0.01 (–0.05, 0.08)
Partner interaction score [β (95% CI )]		0.00 (–0.03, 0.04)		0.02 (–0.02, 0.06)
**a**Referent category.				

In the adjusted complete case analysis, early soy feeding was also significantly associated with higher PSAI scores among girls [® = 2.66 (95% CI: 0.19, 5.12)], whereas primarily breast-feeding was associated with significantly lower scores in boys [® = –0.80 (95% CI: –1.57, –0.03)], compared with early formula feeding (Table 3). Scores were higher among the early soy-fed boys compared with early formula feeding, but this difference was not significant. Associations for early soy exposure derived using multiple imputation were similar but more precise [® (95% CI): girls, 2.83 (0.63, 5.02); boys, 0.90 (–0.88, 2.68)]. No notable association was observed in the late soy exposure group for either sex.

**Table 3:** Adjusted*a* difference in mean PSAI scores for boys and girls from complete case analysis.

β-Coefficient (95% CI)		
Covariate	Boys (*n* = 3,010)	Girls (*n* = 2,823)
Infant feeding method				
Early formula		0		0
Early soy		1.18 (–0.73, 3.09)		2.66 (0.19, 5.12)
Late soy		–0.53 (–1.98, 0.91)		–0.03 (–1.78, 1.71)
Primarily breast-fed		–0.80 (–1.57, –0.03)		–0.26 (–1.10, 0.58)
Presence of older brother				
No		0		0
Yes (≥ 1)		2.06 (1.39, 2.73)		3.24 (2.48, 4.00)
Presence of older sister				
No		0		0
Yes (≥ 1)		–2.75 (–3.42, –2.08)		–1.89 (–2.65, –1.13)
Regular child care attendance				
No		0		0
Yes		0.32 (–0.31, 0.96)		0.41 (–0.29, 1.10)
Prenatal smoking				
No		0		0
Yes		1.42 (0.57, 2.27)		2.08 (1.11, 3.05)
Maternal education				
University degree		0		0
Advanced level		1.29 (0.31, 2.27)		–1.31 (–2.37, –0.24)
Ordinary level		2.01 (1.05, 2.97)		–2.59 (–3.65, –1.53)
Vocational		1.71 (0.41, 3.00)		–2.74 (–4.17, –1.30)
CSE/none		2.48 (1.22, 3.74)		–2.21 (–3.61, –0.81)
Age at PSAI assessment		0.13 (–0.27, 0.53)		–0.64 (–1.10, –0.18)
Maternal age		0.06 (–0.01, 0.13)		0.10 (0.01, 0.18)
Maternal interaction score		0.03 (–0.03, 0.10)		0.02 (–0.06, 0.09)
Partner interaction score		0.01 (–0.03, 0.05)		0.03 (–0.02, 0.07)
**a**Adjusted model includes all variables shown here.				

For both sexes, the adjusted mean difference in PSAI score was higher if an older brother was present in the home or if the mother smoked prenatally and lower if an older sister was present. With increasing maternal education, scores generally decreased for boys and increased for girls, suggesting that highly educated mothers were more likely to report a mixture of masculine and feminine traits, regardless of their child’s sex, than were less educated mothers. Age at assessment and maternal age were associated with lower and higher scores among girls, respectively. These findings are generally similar to previous reports on this cohort (Hines et al. 2002b; Rust et al. 2000).

In post hoc analyses, mean PSAI scores [mean (95% CI)] at 30 and 57 months were higher in early soy-fed girls [42.7 (40.3, 45.1) and 36.9 (34.4, 39.5), respectively] compared with the early formula referent [40.5 (40.2, 40.9) and 35.2 (34.8, 35.6), respectively]. However, the mean difference between these exposure groups weakened at both time points after adjustment for age at assessment, maternal education, maternal age, presence of older brother, presence of older sister, and prenatal smoking [® (95% CI): 30 months, 0.55 (–1.58, 2.69); 57 months, 0.64 (–1.87, 3.15)]. Associations between PSAI scores at 30 and 57 months and primarily breast-feeding among boys were consistent with the association at 42 months [® (95% CI): 30 months, –0.89 (–1.60, –0.19); 57 months, –1.09 (–1.91, –0.26)].

## Discussion

Early-life soy exposure was associated with a slightly higher PSAI score among girls at 42 months of age, whereas soy exposure and PSAI scores were positively but not significantly associated in boys. The increase in PSAI scores among early soy-exposed girls was small and did not place them outside the range of normal female behavior, but it was robust to adjustment for strong predictors of behavior, including the presence of an older brother or sister. In fact, the effect size observed in soy-exposed girls was similar to that observed in girls with older brothers, although much less precise.

Among early soy-fed girls, associations with the 30- and 57-month assessments were weaker than our primary findings for the 42-month assessment, suggesting that our primary findings may be overestimated. However, because boys’ and girls’ behaviors become increasingly sex specific with age (Golombok et al. 2008), it is also possible that the influence of an environmental factor such as soy may become more or less important over time. These additional analyses encourage cautious interpretation of our findings but do not invalidate them.

We also observed that boys who were primarily breast-fed had lower PSAI scores at all time points than did boys fed nonsoy formula. Duration of breast-feeding has previously been associated with feminized play behavior in girls but not in boys (Sandberg et al. 2003). In both sexes, an inconsistent association between breast-feeding and externalizing behavior has also been reported (Kramer et al. 2008; Samarakkody et al. 2011). However, studies of breast-feeding and child development are often met with issues of unmeasured confounding related to differences between breast-feeding and formula-feeding mothers that are typically greater than differences between users of different types of formula. Accordingly, the relationship observed in our study may be confounded by unmeasured factors associated with a mother’s choice to breast-feed and her attitudes toward gender roles in male children. Therefore, despite a consistent association over time, this finding warrants further study before meaningful conclusions can be drawn.

Our findings among early soy-exposed girls are supported by a modest literature. In female rats, the volume of the sexually dimorphic nucleus of the medial preoptic area of the hypothalamus has been shown to increase (Faber and Hughes 1991, 1993), and reproductive posturing behaviors have been altered after neonatal exposure to high levels of genistein (Kouki et al. 2003). Other estrogenic compounds, such as diethylstilbestrol, have also been shown to induce limited masculinization of behavior in female primates, but not humans, after prenatal exposure (Goy and Deputte 1996; Lish et al. 1991). In addition, sex differences in response to soy exposure have been suggested in a recent study demonstrating that preschool-age girls excrete increasing levels of urinary testosterone, whereas boys excrete decreasing levels of urinary estrogens, with increasing dietary soy consumption (Wada et al. 2011).

The susceptibility of neurodevelopmental processes to postnatal endocrine disruption is unclear. Visual preference for groups (i.e., multiple individuals) over solitary individuals, a male-typical trait, has been associated with postnatal testosterone concentrations in male infants, suggesting that some gender-typical preferences may be susceptible to postnatal endocrine disruption (Alexander et al. 2009). However, to our knowledge, no studies to date have reported an association between postnatal endocrine activity and visual, social, or other gender-typical preferences in girls, and potential mechanistic explanations are sparse.

In this study, soy product use in early and late infancy was characterized using longitudinal exposure assessment. However, soy users were not necessarily exclusively fed soy products at any time, and we could not characterize the relative dose of soy. Thus, we could not assess a dose–response relationship between soy feeding and PSAI score or account for other dietary exposures to soy isoflavones. Improved characterization of early-life soy exposure, either through more detailed questionnaires or isoflavone biomarkers, should be applied in future studies.

## Conclusions

Our study suggests that early-life exposure to soy products may subtly reduce female-typical play behaviors in girls at 42 months of age. Given the low prevalence of soy use in this study sample, associations between soy exposure and PSAI score were imprecise, and results should be interpreted cautiously. An association between breast-feeding and play behavior in boys was also observed but may be heavily confounded by unmeasured lifestyle factors, given the broad differences that generally persist between breast-feeding and formula-feeding mothers. The associations observed here were modest, and the mean PSAI score for all exposure groups was still within the range of normal behavior for each sex. Replication of these findings in other populations is needed, particularly in ones with more prevalent SBF use.
